# Morphological, Physicochemical and FTIR Spectroscopic Properties of Bee Pollen Loads from Different Botanical Origin

**DOI:** 10.3390/molecules24213974

**Published:** 2019-11-03

**Authors:** Sara Castiglioni, Paola Astolfi, Carla Conti, Elga Monaci, Mariassunta Stefano, Patricia Carloni

**Affiliations:** 1 Department of Agricultural, Food and Environmental Sciences - D3A, Università Politecnica delle Marche, Via Brecce Bianche, I-60131 Ancona, Italy; s.castiglioni@univpm.it (S.C.); e.monaci@univpm.it (E.M.); 2 Department of Materials, Environmental Sciences and Urban Planning - SIMAU, Università Politecnica delle Marche, Via Brecce Bianche, I-60131 Ancona, Italy; p.astolfi@univpm.it (P.A.); c.conti@univpm.it (C.C.); 3 ASSAM, Agenzia per i Servizi nel Settore Agroalimentare delle Marche, Centro Agrochimico Regionale, I-60035 Jesi (AN), Italy; stefano_mariassunta@assam.marche.it

**Keywords:** bee pollen, color, FTIR-ATR, protein, morphology

## Abstract

Bee pollen loads generally have a homogeneous and monospecific pollen content and assume a typical form and color, due to the typical bee foraging habits, thus having a typical composition related to the botanical origin. The present study aims to characterize bee pollen loads belonging to different botanical species using morphological, spectroscopic and color properties and to find relationships between these variables. IR spectra analysis allowed to have a reliable picture of the components present in the different samples; color and granulometry permits a visual identification of pollen load belonging to different species. Multivariate analysis enabled differentiation among the botanical origin of most of the bee pollen samples, grouping them according to the family and the genus and confirming the possibility to use IR and color measurements for the evaluative analysis and classification of bee pollen samples, to promote the consumption of this bee product as functional food.

## 1. Introduction

Bee pollen is one of the main protein sources of the hive and is collected and processed by worker bees that form loads with a homogeneous and monospecific pollen content and with a typical form and color: in fact, usually bees inspect in a short period an area around the beehive where a single flowering is prevalent [1]. Therefore, the characterization of collected bee pollen can be useful to individuate species of flora preferentially explored by bees and to extend knowledge for the apiculture development [2]. In addition, it is possible to obtain mono- or poly-pollen species loads according to the season, to the timing of collection and to the post-collection management [3].

In the last years, there has been considerable research on different types of honeybee products, whose composition is significantly influenced by the presence of different types of pollen [4,5] and geographical origin [6]. However, limited studies have been carried out on the physicochemical properties of the Italian bee pollen or, even more importantly, on bee pollen of different botanical origins [3,7,8]. Several studies have analyzed the nutraceutical properties of pollen showing its beneficial effects to human health associated to its antioxidants and polyphenolic content [9] and correlated to the botanical species [8].

In this perspective the present study aims to characterize the botanical, morphological, spectroscopic and color properties of several bee pollen samples belonging to different floras and collected in the Marche region (Italy) in order to find relationships and be able to investigate and correlate them.

## 2. Results

**Figure 1:**
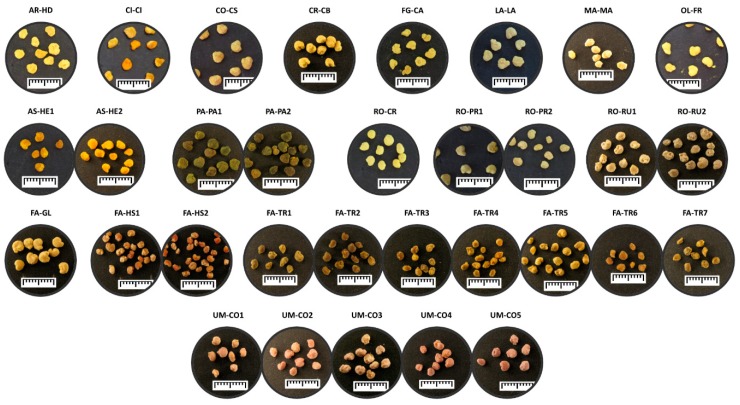
Bee pollen loads visual appearance. Samples were labelled with letters identifying the family and the genus as reported in Table 1. The segment represents 1 cm width.

A total of 32 separated pollen samples were analyzed (Figure 1) in order to identify the frequency of each pollen class: predominant pollen, secondary pollen, and important minor pollen (Table 1). Regarding the floral origin of the analyzed samples, *Trifolium* (FA-TR) and *Coriandrum* (UM-CO) were the predominant genera with the higher number of samples. Other important genera characterizing as predominant pollen, observed as two pollen samples, are *Helianthus* (AS-HE), *Hedysarum* (FA-HS), *Papaver* (PA-PA), *Prunus* (RO-PR) and *Rubus* (RO-RU). These results are in agreement with the predominant species spread in the studied region that the bees usually visit to produce the hive’s products [6].

### 2.1. Palynological Analysis

Palynological characteristics of the samples are reported in Table 1 where percentages of each pollen type are reported for each sample. The analysis, in some cases, did not allow the determination of the botanical species, because the similarity between the pollens of the different species belonging to the same genus does not make the identification possible: in these cases, only the genus (e.g., *Magnolia*) or the pollinic type (e.g., *Prunus* form) is reported. In some other cases, the pollens of different genera belonging to the same family, are very similar and the botanical genus was determined crossing palynological analysis with beekeepers’ indications. Samples are classified depending on the genus and labelled with letters identifying the family and the genus of the predominant pollen.

Table 1 shows that twenty-four samples have a predominance of a pollen type greater than 90%; five samples show the main pollen type in a percentage between 70% and 89% and only in three samples is the predominant pollen type between 55% and 69%. All the samples show a predominance of a pollen type greater than 55%.

### 2.2. Instrumental Color Measurement

The pollen loads’ color is a physicochemical parameter which plays a crucial role in characterizing the samples. The colorimetric characteristics of pollen loads of different floral origin are reported in Table 2.

The results, measured by CIE L*a*b* method, show a high and consistent variability among the different types of loads. In particular, *Trifolium alexandrinum* L. (FA-TR2) and *Papaver rhoeas* L. (PA-PA1) pollens are the darkest, with L* values of 30.8 and 28.6, respectively, lower than 40. The L* value increased in the other pollens up to around 67 in *Magnolia* (MA-MA) and *Hedera helix* L. (AR-HD) pollen. The a* (red-green) parameter of the pollen loads varied from –1.9 (*Papaver rhoeas* L. PA-PA1) to 22.3 (*Helianthus annuus* L. AS-HE2) and the b* (yellow-blue) one from 12.0 (*Coriandrum sativum* L. UM-CO5) to 69.4 (*Helianthus annuus* L. AS-HE2). The b* and a* values were in a similar range as those reported by other authors [10]. The a* (red-green) and b* (yellow-blue) parameters of the pollen loads may be interpreted as a reliable index of the richness in pigments with antioxidant activity [8] and of a different minerals’ concentration that is related to the botanical origin [11].

Color coordinates of pollen loads were also used as variables for the multivariate statistical elaboration of chemical–physical data of pollen loads.

### 2.3. Pollen Load Size Distribution

In Table 2 the mass percentage of pollen loads sized in one of the six groups (diameters of <1400, 1690–1400, 2000–1690, 2400–2000, 2800–2400 and >2800 µm) is reported for each pollen sample.

The largest pollen loads are those of *Gleditsia triacanthos* L. (FA-GL) that differ from all other pollens, containing 45% of loads with a diameter >2800 µm and 49% between 2400 and 2800 µm. Slightly smaller than the previous ones are the loads of *Cornus sanguinea* L. (CO-CS), *Cistus* (CI-CI), *Lamium* (LA-LA) and *Prunus* (RO-PR) whose sizes of most of the loads (89%, 72%, 67% and 63%, respectively) have a diameter larger than 2400 µm with only a small percentage with a diameter greater than 2800 µm. Samples of *Papaver rhoeas* L. (PA-PA), *Rubus ulmifolius* S. (RO-RU), *Crataegus monogyna* Jacq. (RO-CR), *Coriandrum sativum* L. (UM-CO), *Hedera helix* L. (AR-HD), *Brassica* (CR-CB), *Castanea sativa* Miller (FG-CA) and *Fraxinus ornus* L. (OL-FR) contain most of the loads with a diameter between 2000 µm and 2800 µm, while *Helianthus annuus* L. (AS-HE), most of *Trifolium alexandrinum* L. (FA-TR) and *Magnolia* (MA-MA) samples have a high percentage of the loads with a diameter between 1690 µm and 2400 µm. The smallest pollen loads are those of *Hedysarum coronarium* L. (FA-HS) having mainly a diameter between 1400 µm and 2000 µm.

### 2.4. Protein Determination

In Table 2 the percentage (%) of proteins in pollen samples is reported: bee pollens show a high amount of proteins that varies considerably according to the botanical species (from 13.6% in *Helianthus annuus* L. AS-HE2 to 40.7% in *Hedysarum coronarium* L. FA-HS1). Most of these results are in accordance with previous studies that report a low percentage of protein in *Helianthus* [7,12,13] and higher ones in *Castanea* [7,14], *Magnolia* [7], *Lamium* [15], *Papaver* [13,15], *Rubus* [7,15,16] and *Prunus* [15]. Further, *Coriandrum sativum* L. samples (UM-COs) show a relatively low amount of protein (from 18.5% to 23.3%), while the amount in *Trifolium alexandrinum* L. samples (FA-TRs) is higher than literature reports [15,16] and shows a considerable percentage variability (from 23.5% to 33.0%).

### 2.5. FTIR-ATR Spectral Characterization

**Figure 2:**
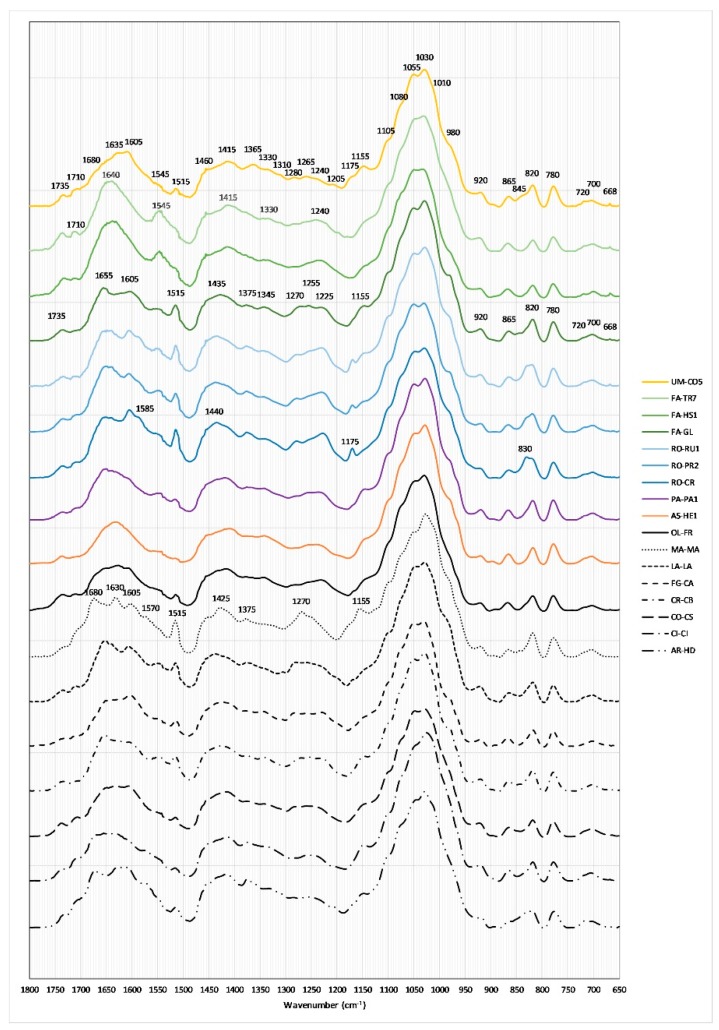
FTIR-ATR spectra of bee pollen samples. Only one sample representative of each genus is reported.

The FTIR-ATR spectra of pollens belonging to different genera as shown in Figure 2 were only a sample representative of each genus reported. Main constituents of bee pollen are proteins, saccharides and lipids; IR spectra of the pollen samples have similar characteristic peaks [17,18,19], but several peculiarities can be found in dissimilar pollens due to little differences in chemical compositions. The meaningful IR peaks of bee pollens are shown in Table 3.

All pollens show a broad band at 3260 cm^−1^ corresponding to O–H stretching vibration from water, and two strong peaks at 2925 and 2855 cm^−1^, with some difference for PA-PA and AS-HE which have an additional peak at 2930 cm^−1^. These peaks are assigned to the C–H stretching vibrations of cellulose and lipids. Because in this zone the differences between the various pollens are very small this region was not shown and only the spectral region 1800–650 cm^−1^ was considered. All pollen samples show peaks or shoulders in the range 1800–1500 cm^−1^ due to C=O groups and to the C=C stretching vibrations of phenolic acids (see Table 3). In this region it can be pointed out that some samples show intense peaks at 1680 (CI-CI, MA-MA, AR-HD) and/or at 1630 cm^−1^ (UM-CO, AS-HE, MA-MA, AR-HD) but not the peak at 1660 cm^−1^ (MA-MA, AR-HD). The broad peaks between 1440 and 1390 cm^−1^ result from C–H deformation vibration of lipids and cellulose, those between 1375 and 1345 cm^−1^ from the CH_3_ bending vibrations in aliphatic groups, while a shoulder or a convolution of peaks around 1340 and 1200 cm^−1^ can be assigned to the amide III (the in-phase combination of N–H deformation and C–N stretching vibrations). It is noteworthy that in this zone are observed the characteristic patterns of MA-MA with a strong peak at 1270 cm^−1^, those of the three RO samples with two peaks at 1280 and 1225 cm^−1^ and those of FA-GL and CR-CB with three peaks at 1275, 1255 and 1225 cm^−1^. The 1200–500 cm^−1^ range is the so-called fingerprint region, where a strong peak (1200–950 cm^−1^) with shoulders at 1105, 1080, 1055, 1030, 1010 and 980 cm^−1^ is observed for all samples, corresponding to the C–O, C–N and C–C stretching vibrations of sugar and proteins. In addition, in this region, the three RO samples show a peak at 1170 cm^−1^ and the MA-MA one at 1155 cm^−1^. Bands at 920, 865, 820, 780 and 700 cm^−1^ are characteristic for all pollen samples and can be assigned to saccharides. Again, RO samples show a typical peak at 720 cm^−1^ together with AR-HD. The bands below 700 cm^−1^ could be assigned to various skeletal vibrations: in this region FA-GL, FA-HS, FA-TR, RO-RU, UM-CO, CI-CI, MA-MA and CR-CB show a small but typical peak at 668 cm^−1^.

IR spectra analysis points out that the composition of saccharides, proteins, lipids and polyphenols is different for dissimilar bee pollens; with this perspective the spectral region 1800–650 cm^−1^ can be used to perform multivariate analysis, as already reported [18].

## 3. Discussion

There are several reports focused on the possibility to characterize the botanical origin of bee products using multivariate statistics on spectroscopic data like FTIR. Kasprzyk et al. in 2018 [18] investigated the floral origin of bee pollens using a sequence of chemometric procedures (DA, HCA, etc.) applied on parameters obtained from FTIR spectra to confirm the floral origin of rape pollen; Svecniak et al. in 2015 [20] explored the possible clustering of unifloral honeys belonging to different pollens using principal component analysis; in addition Bleha et al. in 2019 [17] characterized six unifloral bee pollens of various botanical origins by morphometry, SEM, CIE L*a*b* color parameters and FTIR spectroscopy. However, in the literature there are no reports on the use of FTIR data together with those of color and granulometry to identify the botanical origin of bee pollen loads. In previous papers we reported that pollens’ floral origin characterizes the phenolic profile and the color [4,8] of the bee products, and since it is well known [21] that FTIR spectroscopy is able to assess and quantify phenolic compounds in foods, a multivariate analysis was applied to morphology characteristics, color parameters and IR spectroscopic data of bee pollen loads to have a more simplified view of their relationship with the botanical origin.

### 3.1. Cluster Analysis

First, a hierarchical cluster analysis (HCA) was performed to verify if the data structure would be able to identify groups among the pollen samples.

For the analysis, the data set comprised color coordinates (L*, a* and b*), granulometry and IR data pre-processed with standard normal variable (SNV) and second derivative from 1800 to 660 cm^−1^, for all pollen samples [18]. The complete linkage algorithm was applied using the Euclidean distance to space the cluster.

**Figure 3:**
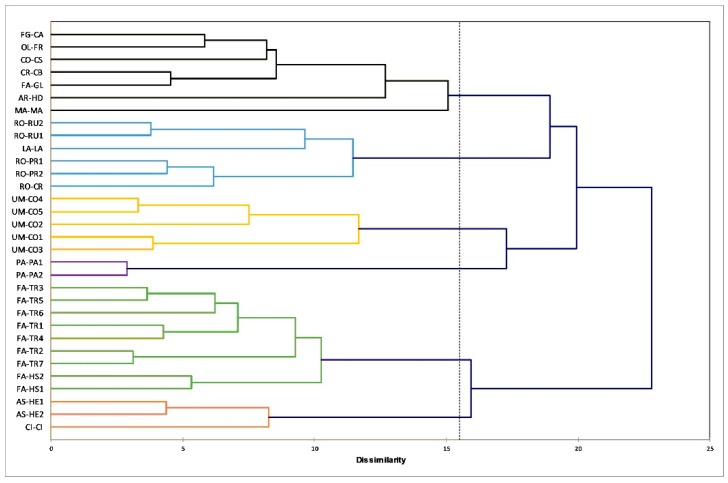
Dendrogram obtained by cluster analysis performed applying the complete linkage algorithm (Euclidean distance) on the data set incorporating all pollen samples and color coordinates (L*, a* and b*), granulometry and second derivative IR data (1800–650 cm^−1^) as variables.

The result obtained by cluster analysis, presented as a dendrogram (Figure 3), shows the presence of eight pollen clusters. Fabaceae (except *Gleditsia*), Umbelliferae, Rosaceae, Papaveraceae and Asteraceae pollen samples are each grouped in their own clusters, while the other samples belonging to different families are grouped together (FG-CA, OL-FR, CO-CS, CR-CB, FA-GL and AR-HD, MA-MA) or are comprised in other groups (LA-LA and CI-CI). The possibility to group most of the pollen samples into separate clusters corresponding to the families indicates that morphology characteristic, color parameters and IR spectra contain useful information for classifying the samples.

### 3.2. Principal Component Analysis (PCA)

PCA permits the extraction of systematic variations in data set and could be used for the classification of samples and interpretation of their differences. In this study, it was employed to classify pollen samples from their IR spectra together with data describing the morphology of the samples.

**Figure 4:**
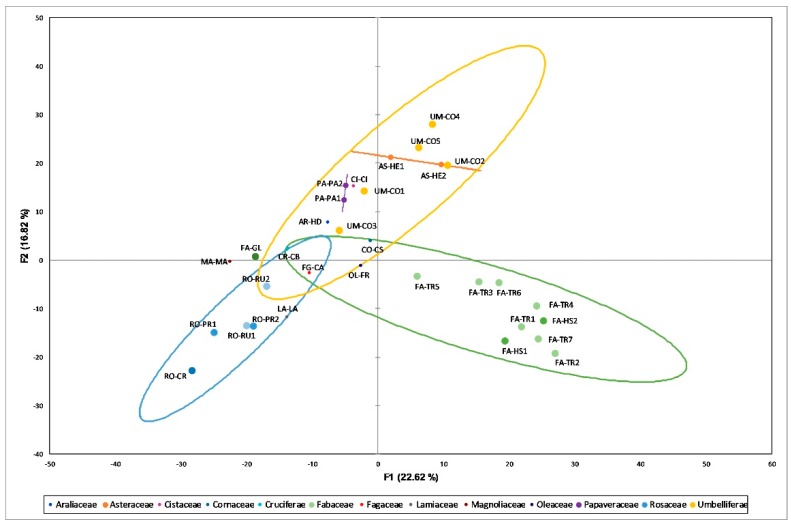
Score plot of the first two components obtained by PCA performed on data set incorporating all pollen samples and color coordinates (L*, a* and b*), granulometry and second derivative IR data (1800–650 cm^−1^) as variables. The botanical origin (family) was used as passive variable to represent ellipses corresponding to 80% confidence interval for a bivariate normal distribution with the same means and the same covariance matrix as the factor scores for each group.

The PCA was performed on the same data set used for HCA and provided good results in order to clearly identify the botanical origin of samples. A PCA-explained variance of 70.4% can be found by using five principal components with an eigenvalue >1. The first two coordinates PC1 and PC2 mainly contain information described by IR spectra and explain 39.44% of the total variance (22.62% PC1 and 16.82% PC2) with PC1 also containing color and granulometry information. IR absorptions mainly contributing to the factors are 1528, 1510, 1457, 1435 and 1395 cm^−1^ for PC1 and 1492, 1355, 1152, 795, 775 and 750 cm^−1^ for PC2. Figure 4 shows the score plot of the first two PCs of the model, which display the aggregation of samples characterized by a relatively high number of specimens in three different groups in accordance with their floral families. Fabaceae samples (FA-GL, FA-HSs and FA-TRs samples) are located in the lower right area of the graph with the *Gleditsia* sample (FA-GL) shifted more to the left and located rather far from the others. This result, which agrees also with granulometry (bigger), color (lighter) and antioxidant activity [8], could be due to the fact that despite belonging to the same family, *Gleditsia* is an arboreal genus while *Trifolium* and *Hedysarum* are herbaceous ones. On the contrary, samples belonging to the Rosaceae family (RO-CR, RO-PRs and RO-RUs) with their lighter color are located in the lower left area of the graph: in this case all the samples belonging to the same family but to different genera are in the same area and couple together. On the upper area it is possible to find Umbrelliferae samples (UM-COs) characterized by a pink color and a medium granulometry.

## 4. Material and Methods

### 4.1. Pollen Samples

Pollen samples were collected in 2014 and 2015 in the Marche region, by professional beekeepers from beehives equipped with bottom-fitted pollen traps, located near areas where bee plants were flowering. The pollen loads were harvested every 2–3 days during the blossom season to minimize changes in the diversity of the available pollen sources in the area. These factors allowed to obtain samples characterized by low palynological diversity. Pollen were cleaned from impurities and kept in plastic bags at −21 °C until the delivery to the laboratory.

Following the beekeeper’s indication, pollen samples from the same area and flora source were then pooled and dried at 35 °C for three days to reach a moisture content lower than 10%. Finally, the pollen loads of each batch were manually separated according to color, shape and size allowing to get 32 pollen samples. The pollen samples were stored in the dark at room temperature.

Palynological analysis crossed with beekeepers’ indications allowed to classify bee pollen samples in 13 botanical families and 17 genera according to the prevalent pollen in each sample. Samples were labelled with letters identifying the family and the genus (Table 1).

### 4.2. Palynological Analysis

Two grams of each pollen sample was vigorously stirred in 15 mL of water for 30 min. The suspension was further diluted with 45 mL of water and stirred again before pollen analysis.

Three small drops of the well-mixed pollen grain suspension were applied on a microscope slide, dried on a heating plate, and a few drops of glycerin jelly were added before covering it with the cover slide. Pollen grain counts were done under the microscope.

Regarding the botanical identification of the samples, in some cases it was not possible to identify the single botanical species but only the genus (see for example the *Magnolia*), because of the similarity between the pollen types of the different species belonging to the same genus. In other cases, the level of similarity between species obliges the grouping of pollen by “pollen types”, i.e., groupings based on the similarity of the pollen, which does not necessarily correspond to the systematic division used by botanists. In these cases, a note should be added to the scientific name indicating that the term is used with a broader meaning than the normal one generally used. For this purpose, the following terminologies can be used:

- group (gr.): when referring to a specific species (e.g., *Trifolium repens* gr.) and one is certain of the belonging of the unknown pollen to the same botanical genus or to a related genus;
- form (f.): when referring to a botanical genus (e.g., *Eucalyptus* f., or “M” form for the Labiatae) and one is certain of belonging to the same botanical family;
- type (t.): when the unknown pollen has a similarity with another known pollen but is not possible to state that it belongs to the same botanical family (we can refer to a genus or a family, e.g., Moraceae t.) [22].

The results were expressed as a percentage of the pollen type (Table 1).

### 4.3. Visual Characterization

A camera was used to take images from each of the pollen load samples. This set of images was used to identify the different pollen types by macroscopic morphology and color.

The morphology of the bee pollen loads of each genus was represented in Figure 1.

### 4.4. Instrumental Color Measurement

The colors of the surface of pollen loads and of the finely ground pollen samples were determined using a Konica Minolta CR-400 (Konica Minolta, Sensing Inc., Osaka, Japan) chroma meter equipped with a D65 illuminant and operating with CIE L*a*b* (L*: 0 to 100, a*: −green to +red, and b*: −blue to +yellow) color space. Calibration was performed with the white color calibration tile (Y = 86.6, x = 0.3188, y = 0.3364) prior to the measurements.

Approximately 3 g of each pollen sample was poured into a sample holder and three readings were taken from each sample surface. The results of color coordinates are expressed as mean values from the three independent experiments (*n* = 3) and are reported in Table 2.

### 4.5. Pollen Load Size Distribution

Pollen load size distribution was measured by sieves analysis. 10 g of pollen was loaded into a series of 20 cm-diameter sieve trays (from top to bottom: 2800, 2400, 2000, 1690 and 1400 µm hole diameters). After shaking the sample in sieves for two minutes, particles retained on the sieves were collected and weighed. The weight of each solid fraction was compared to the weight of the total solid to obtain the mass percentage of solid held by each plate and to classify the pollen load into six groups [23] (Table 2).

### 4.6. Moisture Determination

The moisture of the samples was determined after drying using the internal method used by ASSAM (Agenzia per i Servizi nel Settore Agroalimentare delle Marche). Two grams of pollen was ground in a mortar to obtain a homogeneous powder which was spread in a thin and homogeneous layer. The sample was inserted into a thermobalance and was progressively heated until it reached the temperature of 90 °C in 3 min, which was kept for 40 min. The thermobalance automatically calculates the weight loss. The results were expressed as moisture percentage and were used to calculate the protein content of the samples,

### 4.7. Total Protein Content (%)

Total nitrogen content was determined through the Dumas method (dry combustion method). Pollen samples were weighed (4.0 ± 0.1 mg) into small tin capsules and heated in a purified O_2_ stream to a temperature of 1000 ± 10 °C to promote the full oxidation of organic N. The analysis was performed by using a CHNS-O Elemental Analyzer (EA 1110-CHNS-O, CE Instruments) equipped with an oxidation (chromium oxide)/reduction (pellets of pure copper) analytical column. The running time was set at 250 sec and acetanilide (C 71.09%; N 10.36%; H 6.71%; O 11.84%) was used as standard molecule to calibrate the instrument. Factor 6.25 was used to covert the total nitrogen into proteins. Protein content was expressed as protein percentage (Table 2) considering moisture content of the samples.

### 4.8. FTIR-ATR Data Acquisition and Data Processing

Before spectral acquisition, approximately 1.0 g of representative samples was taken and very well mixed and ground in a mortar to obtain homogeneous powders; five little portions were used for the infrared analysis. The pollen spectra were acquired by a Perkin-Elmer FTIR spectrometer (Spectrum GXI, Waltham, MA, USA) in attenuated total reflection (ATR) mode, by using U_ATR DuraSamplIR accessory (Smiths Detection Inc., Danbury, CT, USA) (spectral resolution of 4 cm^−1^, spectral range 4000–650 cm^−1^, 64 scans). A background spectrum was collected before each acquisition and the ATR element was thoroughly washed with bi-distilled water and dried with a soft tissue. The experiments were carried out at room temperature: i.e., 25 ± 2 °C.

For all the samples, the average spectra were calculated (*n* = 5). Raw FTIR-ATR spectra were converted in absorbance and interpolated in the 1800–650 cm^−1^ spectral range. To optimize the calibration accuracy, spectra were submitted to baseline and multiplicative scatter correction, (MSC).

### 4.9. Multivariate Analysis

Chemometric examination of FTIR-ATR spectra was performed on spectral data preprocessed with standard normal variable (SNV) and second derivative (Gonzalez-Martin 2007). Spectral data treatment was performed using the Pirouette^®^ 4.5 software (Infometrix, Bothell, WA, USA). The resulting spectra were processed using multivariate chemometric techniques involving cluster analysis (CA) and principal component analysis (PCA). All multivariate statistical treatments were performed using XLSTAT software (Addinsoft SARL, Paris, France).

## 5. Conclusions

Different types of bee pollen, obtained from Marche region, were analyzed providing a morphology and spectroscopic characterization of some Italian bee pollen types.

All the evaluated parameters were used to characterize and differentiate the various bee pollens. IR spectra analysis allowed to have a more reliable picture of the components present in the different samples since saccharides, proteins, lipids and polyphenols can give a different spectral characterization of samples.

Multivariate analysis of color, granulometry and IR data enabled differentiation among the botanical origin of most of the bee pollen samples, grouping them belonging to the family and the genus and confirming the validity of such analysis for the characterization and classification of bee pollen samples. These results suggest the possibility to use IR measurements for the analysis and classification of bee pollen samples according to their origin with easy and low-cost instrumental techniques which are rapid and can be used routinely in non-destructive daily analysis.

Finally, monofloral bee pollens were characterized, thus promoting the production and the consumption of these bee products.

## Figures and Tables

**Table 1:** Palynological characteristics of the bee pollen samples. Identification of pollen type, frequency, family and genus are provided. The botanical species was determined using the knowledge on territory’s flora, the cultivation maps and beekeepers’ indications.

Sample	Family	Genus	Determined Species and Predominance	Palynological Analysis
AR-HD	Araliaceae	*Hedera*	*H. helix* L. 100%	*H. helix* L. 100%
CI-CI	Cistaceae	*Cistus*		*Cistus incanus/creticus* 86%, *Castanea sativa* Miller 10%
CO-CS	Cornaceae	*Cornus*	*C. sanguinea* L. 100%	*C. sanguinea* L. 100%
CR-CB	Cruciferae	*Brassica*		Cruciferae Brassica type 100%
FG-CA	Fagaceae	*Castanea*	*C. sativa* Miller 100%	*C. sativa* Miller 100%
LA-LA	Lamiaceae	*Lamium*		Lamiaceae L form 100%
MA-MA	Magnoliaceae	*Magnolia*		*Magnolia* 69%, *Plantago* 12%, Umbelliferae 7%, Asteraceae S Form (*Carduus*) 5%
OL-FR	Oleaceae	*Fraxinus*	*F. ornus* L. 90%	F. ornus L. 90%, Quercus robur gr. 10%
AS-HE1	Asteraceae	*Helianthus*	*H. annuus* L. 45%	Asteraceae T form (*Taraxacum* and *Cichorium*) 55%, *H. annuus* L. 45%
AS-HE2	Asteraceae	*Helianthus*	*H. annuus* L. 77%	*H. annuus* L. 77%, Asteraceae T form (*Taraxacum* and *Cichorium*) 23%
PA-PA1	Papaveraceae	*Papaver*	*P. rhoeas* L. 100%	*P. rhoeas* L. 100%
PA-PA2	Papaveraceae	*Papaver*	*P. rhoeas* L. 100%	*P. rhoeas* L. 100%
RO-CR	Rosaceae	*Crataegus*	*C. monogyna* Jacq. 70%	*C. monogyna* Jacq. 70%, *Salix* 20%, Quercus robur gr. 10%
RO-PR1	Rosaceae	*Prunus*		Prunus f. 100%
RO-PR2	Rosaceae	*Prunus*		Prunus f. 80%, *Salix* 18%
RO-RU1	Rosaceae	*Rubus*	*R. ulmifolius* Schott 100%	*R. ulmifolius* Schott 100%
RO-RU2	Rosaceae	*Rubus*	*R. ulmifolius* Schott 100%	*R. ulmifolius* Schott 100%
FA-GL	Fabaceae	*Gleditsia*	*G. triacanthos* L. 100%	*G. triacanthos* L. 100%
FA-HS1	Fabaceae	*Hedysarum*	*H. coronarium* L. 100%	*H. coronarium* L. 100%
FA-HS2	Fabaceae	*Hedysarum*	*H. coronarium* L. 100%	*H. coronarium* L. 100%
FA-TR1	Fabaceae	*Trifolium*	*T. alexandrinum* L. 98%	*T. alexandrinum* L. 98%
FA-TR2	Fabaceae	*Trifolium*	*T. alexandrinum* L. 97%	*T. alexandrinum* L. 97%
FA-TR3	Fabaceae	*Trifolium*	*T. alexandrinum* L. 81%	*T. alexandrinum* L. 81%, *T. repens* L. 17%
FA-TR4	Fabaceae	*Trifolium*	*T. alexandrinum* L. 90%	*T. alexandrinum* L. 90%, *T. repens* L. 10%
FA-TR5	Fabaceae	*Trifolium*	*T. alexandrinum* L. 98%	*T. alexandrinum* L. 98%
FA-TR6	Fabaceae	*Trifolium*	*T. alexandrinum* L. 59%	*T. alexandrinum* L. 59%, *T. repens* L. 17%, *Galega officinalis* L. 6%, *Papaver rhoeas* L. 5%, *Rubus ulmifolius* Schott 5%, Cruciferae 4%, *Lotus corniculatus* L. 3%
FA-TR7	Fabaceae	*Trifolium*	*T. alexandrinum* L. 100%	*T. alexandrinum* L. 100%,
UM-CO1	Umbelliferae	*Coriandrum*	*C. sativum* L. 100%	Daucus and Coriandrum gr. 100%
UM-CO2	Umbelliferae	*Coriandrum*	*C. sativum* L. 100%	Daucus and Coriandrum gr. 100%
UM-CO3	Umbelliferae	*Coriandrum*	*C. sativum* L. 100%	Daucus and Coriandrum gr. 100%
UM-CO4	Umbelliferae	*Coriandrum*	*C. sativum* L. 100%	Daucus and Coriandrum gr. 100%
UM-CO5	Umbelliferae	*Coriandrum*	*C. sativum* L. 100%	Daucus and Coriandrum gr. 100%

**Table 2:** Morphology, instrumental color data, protein content and pollen load size distribution of bee pollen samples.

Sample	Morphology	Color			Protein	Pollen Load Size ^2^ Distribution ^1^					
		L*	a*	b*	Content ^1^	>2800	>2400	>2000	>1690	>1400	<1400
AR-HD	Yellow Ocher	67.6	7.9	47.8	26.7	3	22	58	14	3	0
CI-CI	Yellow Orange	56.5	12.3	48.5	18.3	24	48	26	1	0	0
CO-CS	Ocher Big	56.0	7.0	36.0	17.1	23	66	10	0	0	0
CR-CB	Yellow	60.4	5.8	46.9	23.9	2	30	61	6	1	0
FG-CA	Yellow	63.8	5.7	50.3	23.9	2	20	66	11	1	0
LA-LA	Light Beige	63.5	5.0	29.4	26.2	14	53	30	2	0	0
MA-MA	Dirty White	67.5	6.5	33.9	25.5	0	0	41	31	23	5
OL-FR	Yellow	63.7	6.4	50.1	23.0	2	15	68	12	2	1
AS-HE1	Vivid Orange	55.6	21.6	65.4	14.5	1	10	49	30	9	1
AS-HE2	Vivid Orange	56.8	22.3	69.4	13.6	1	10	58	23	8	1
PA-PA1	Green	28.6	−1.9	18.9	23.8	0	64	25	7	4	0
PA-PA2	Green	29.8	2.0	18.2	24.7	0	29	56	8	7	0
RO-CR	Light Yellow	66.3	3.8	40.8	27.4	2	32	54	9	1	1
RO-PR1	Olive Green	53.3	3.4	23.7	30.2	13	50	32	3	1	0
RO-PR2	Green Beige	57.3	3.0	30.0	25.8	0	5	41	39	14	0
RO-RU1	Light Green	56.0	3.7	18.6	25.5	0	27	50	13	8	0
RO-RU2	Two-Tone Green	50.1	3.9	14.6	24.0	2	16	63	16	3	0
FA-GL	Yellow Hearth	63.9	5.5	39.0	28.8	45	49	6	0	0	0
FA-HS1	Two-Tone Red	51.3	12.7	27.0	40.7	0	1	23	37	36	2
FA-HS2	Red Orange	49.0	16.8	26.9	40.5	0	0	12	32	50	5
FA-TR1	Light Brown	35.3	5.1	22.7	28.6	0	5	54	29	10	0
FA-TR2	Dark Brown	30.8	7.1	21.8	23.5	0	1	54	34	11	0
FA-TR3	Ocher Light Brown	43.3	6.9	29.9	30.8	0	2	48	35	15	0
FA-TR4	Ocher Brown	38.0	8.9	31.3	25.4	0	0	33	46	20	0
FA-TR5	Ocher	49.8	7.4	35.2	23.7	1	6	61	29	4	0
FA-TR6	Brick Red	35.7	10.3	26.1	33.0	1	6	40	37	17	1
FA-TR7	Brown	33.2	7.0	24.3	27.8	0	2	37	48	13	0
UM-CO1	Faded Light Pink	52.9	13.6	19.3	19.8	0	24	55	16	4	0
UM-CO2	Pink	46.9	14.0	12.1	18.5	1	26	63	9	1	0
UM-CO3	Rough Light Pink	57.9	11.7	17.4	22.7	10	35	41	10	2	1
UM-CO4	Vivid Light Pink	50.1	16.9	15.7	23.3	0	24	66	6	3	0
UM-CO5	Opaque Light Pink	51.4	15.1	12.0	18.6	1	23	68	6	1	0

^1^ Protein content and pollen load distribution are expressed in %, ^2^ Pollen load size is expressed in μm.

**Table 3:** Meaningful FTIR-ATR peaks of pollen samples: peak position in terms of wavenumbers (cm^−1^), vibrational mode and chemical assignment.

Peak Position (cm^−1^)	Vibrational Mode	Biochemical Assignments
~3260	Stretching mode of OH	Water
~2924, ~2855	Symmetric and asymmetric stretching modes of CH_2_ moieties	Lipids alkyl chains, cellulose
~1735	Stretching mode of carbonyl moiety	Hemicellulose
~1710	Stretching mode of carboxylic moiety	Carboxylic (shoulder)
~1660	Stretching and bending modes of peptide linkage	Amide I of proteins
~1545	Bending mode of CH_2_ moieties	Amide II (shoulder)
~1515	C=C stretching vibrations	Phenolic acids
~1440–1390	C–H deformation vibration	Lipids and cellulose
~1375–1345	CH_3_ bending vibrations	Aliphatic groups
~1340–1200	In-phase combination of N–H deformation and C–N stretching vibrations	Amide III of proteins
~1030	C–O, C–N and C–C stretching vibrations	Sugar and proteins
~920–700	Vibrational modes of C–OH groups	Saccharides
~708	Bending mode of N–H bond	Amide V of proteins
